# CD82/KAI1和HIF-1α在非小细胞肺癌中的表达及其与血管生成拟态的关系

**DOI:** 10.3779/j.issn.1009-3419.2011.12.04

**Published:** 2011-12-20

**Authors:** 世伍 武, 泽农 承, 岚 俞, 文庆 宋, 仪声 陶

**Affiliations:** 233000 蚌埠，蚌埠医学院第一附属医院病理科，蚌埠医学院病理教研室 Department of Pathology, Bengbu Medical College, the First Affiliated Hospital of Bengbu Medical College, Bengbu 233000, China

**Keywords:** 肺肿瘤, CD82/KAI1, HIF-1α, 血管生成拟态, 预后, Lung neoplasms, CD82/KAI1, HIF-1α, vasculogenic mimicry, Prognosis

## Abstract

**背景与目的:**

新近研究显示血管生成拟态存在于多种高侵袭性肿瘤中, 并与肿瘤细胞的侵袭、转移特性有关, 在形成血管生成拟态的肿瘤中有多种基因表达异常。本研究旨在寻找能预测非小细胞肺癌(non-small cell lung cancer, NSCLC)浸润、转移及术后生存率的指标。

**方法:**

采用免疫组化Elivision^TM^ plus法和特殊组织化学法检测160例NSCLC和20例正常肺组织中缺氧诱导因子-1α(hypoxia inducible factor-1α, HIF-1α)、CD82/KAI1的表达和血管生成拟态(vasculogenic mimicry, VM)情况。

**结果:**

在正常肺组织中HIF-1α、CD82/KAI1的表达率和VM分别为0、95.0%和0, 在NSCLC组织中分别为48.8%、37.5%和36.9%, 差异有统计学意义(*P* < 0.01);其水平与肿瘤细胞分化程度、淋巴结转移、临床分期和术后生存期有关(*P* < 0.01);CD82/KAI1的表达与HIF-1α的表达以及VM呈负相关, HIF-1α的表达水平与VM呈正相关(*P* < 0.05);CD82/KAI1、HIF-1α的表达以及VM均与微血管密度(microvessel density, MVD)有关联性(*P* < 0.01)。*Kaplan-Meier*生存分析表明HIF-1α的过表达和VM均与患者的生存率有关, 阳性的患者生存率明显低于阴性者(*P* < 0.01);而CD82/KAI1阳性表达的患者生存率明显高于阴性者(*P* < 0.01);MVD≥22的5年生存率明显低于MVD < 22的生存率(*P* < 0.01)。多因素分析:pTNM分期、CD82/KAI1、HIF-1α的表达以及VM是影响NSCLC根治术后患者预后的独立因素(*P* < 0.01)。

**结论:**

CD82/KAI1、HIF-1α在NSCLC组织中的表达水平以及VM与肿瘤的分化程度、转移和预后等均有关, CD82/KAI1、HIF-1α和VM联合检测对NSCLC的进展及预后判断有重要意义。

## Abstract (en)

**Background and objective:**

Vasculogenic mimicry (VM), found in many high invasive ability tumors, is associated with tumor invasion and metastasis.Many genes exhibit abnormal levels of expression in these tumors.This study aims to find good markers for predicting the invasion and metastasis of non-small cell lung cancer (NSCLC).

**Methods:**

VM and expression of CD82/KAI1 and HIF-1α were examined via immunohistochemistry and histochemistry of 160 NSCLC and 20 normal lung tissue specimens.

**Results:**

In NSCLC, positive rates of 37.5%, 48.8%, and 36.9% were obtained for CD82/KAI1, HIF-1α, and VM, respectively.In normal lung tissue, positive rates of 95.0%, 0, and 0 were obtained for CD82/KAI1, HIF-1α, and VM, respectively.A significant difference was found between the NSCLC and normal lung groups (*P* < 0.01).VM and the expression levels of CD82/KAI1 and HIF-1α were significantly related to tumor differentiation, lymph node metastasis, clinical staging, and postoperative survival time (*P* < 0.01 for all).A negative correlation was found between the expression levels of CD82/KAI1 and HIF-1α; a similar relationship was observed between CD82/KAI1 and VM.A positive relationship between the expression of HIF-1α and VM was revealed; there was a significant relationship between microvessel density (MVD) and the expression of CD82/KAI1 or HIF-1α or VM.VM and overexpression of HIF-1α were related to poor prognosis:the survival rates were significantly lower in positive patients than in negative patients (both *P* < 0.01).The survival rates of the CD82/KAI1-positive and CD82/KAI1-negative groups were significantly different (*P* < 0.01).The five-year survival rate was significantly different between the group with MVD≥22 and the group with MVD < 22 (*P* < 0.01).pTNM stage, positive expressions of CD82/KAI1 and HIF-1α, and VM were independent prognostic factors of NSCLC (*P* < 0.01).

**Conclusion:**

VM and the expressions of CD82/KAI1 and HIF-1α in NSCLC are related to differentiation, lymph node metastasis, clinical staging, and prognosis.The combined detection of CD82/KAI1, HIF-1α, and VM has an important role in predicting the progression and prognosis of NSCLC.

*CD82*/*KAI1*基因是一个新的肿瘤转移抑制基因, 该基因位于染色体11p11.2, 编码产物为细胞膜糖蛋白, 属于4次跨膜蛋白超家族(transmembrane 4 superfamily, TM4SF)家族成员, 广泛表达于多种组织。其表达与多种肿瘤的演进和预后及胚胎的植入密切相关^[1, 2]^。缺氧诱导因子-1α(hypoxia inducible factor-1α, HIF-1α)是缺氧条件下广泛存在于哺乳动物和人体内的一种转录因子, 是应答缺氧应激的关键基因, 它的活性对肿瘤细胞凋亡、转移及血管生成等生物学行为起着重要作用。血管生成拟态(vasculogenic mimicry, VM)是Yue^[3]^发现的一种独特的肿瘤血液供应方式, 即是一种由肿瘤细胞变形并形成可输送血液的管道结构, 对缓解肿瘤缺氧起重要作用。VM的存在与疾病进展和预后不良相关。本研究通过检测160例非小细胞肺癌(non-small cell lung cancer, NSCLC)病例标本CD82/KAI1、HIF-1α的表达和VM, 旨在寻找能预测NSCLC患者局部浸润、淋巴结转移及术后生存率的指标。

## 1 材料与方法

### 1.1 一般资料

收集蚌埠医学院第一附属医院病理科2003年1月-2005年4月存档石蜡包埋NSCLC组织标本160例(术前未行放、化疗)和正常肺组织标本20例, 所有病例均有完整的临床、病理及随访资料, 入选病例随访至患者死亡或截至2010年4月, 随访时间为3个月-84个月。其中男性130例, 女性30例; 从大体类型看, 中央型120例, 周围型40例; 鳞癌116例(高分化17例, 中分化71例, 低分化28例), 腺癌44例(高分化4例, 中分化32例, 低分化8例)。年龄26岁-82岁, 中位年龄59.6岁, ≥60岁者86例, < 60岁者74例; 肿瘤长径D≥3.0 cm者145例, D < 3.0 cm者15例; 按淋巴结有无转移, 有转移76例, 无转移84例。根据UICC2002版pTNM病理分期标准进行分期, 其中Ⅰ期患者31例, Ⅱ期患者43例, Ⅲ期患者34例, Ⅳ期患者52例。对照组正常肺组织20例取自肺癌肿块 > 5.0 cm的肺组织, 病理HE染色证实为正常肺组织。

### 1.2 试剂

鼠抗人CD82/KAI1单克隆抗体(克隆号:G-2)、鼠抗人HIF-1α单克隆抗体(产品编号:SC-53546)购自Santa Cruz公司; 鼠抗人CD34单克隆抗体(产品编号:MAB-0034, 克隆号QBEnd/10)、Elivision^TM^ plus试剂盒以及DAB显色试剂盒均购自福州迈新生物技术开发公司。PAS染色液为蚌埠医学院第一附属医院病理科配制。

### 1.3 实验方法

#### 1.3.1 采用免疫组织化学Elivision^TM^ plus法

将石蜡标本以4 μm厚连续切片、烤干, 于二甲苯溶液及不同浓度的乙醇中脱蜡至水洗。免疫组化染色操作步骤按试剂盒说明书进行。采用已知阳性片作对照, 以PBS液代替一抗作空白对照。

#### 1.3.2 CD34和PAS套染

CD34染色, DAB显色后, 流水冲洗1 min终止显色反应, PAS染色按照《临床技术操作规范—病理学分册》说明进行。

#### 1.3.3 微血管密度(microvessel density, MVD)计数

CD34主要表达在血管内皮细胞的细胞浆和胞膜, 从而可以通过CD34的阳性表达来进行MVD计数, 参照修改过的Weidner^[4]^法来计数。

### 1.4 结果判定

CD34和CD82/KAI1均以细胞膜和细胞浆出现棕黄色颗粒为阳性; HIF-1α蛋白以细胞核内和胞浆有棕黄色细颗粒为阳性。采取二次计分法:每例标本随机计数5个高倍视野(×400), 计数每个高倍视野中阳性细胞所占百分比并计分。首先将染色强度计分:0分为无色, 1分为淡黄色, 2分为棕黄色, 3分为棕褐色。再将阳性细胞百分比计分, 0分为阴性, 1分为阳性细胞为 < 10%, 2分为11%-50%, 3分为51%-75%, 4分为 > 75%。用染色强度得分和细胞数得分的乘积作为判断表达结果, 若积分≤1为阴性, > 1为阳性。免疫组化结果由高年资病理医师读片评定。

### 1.5 统计分析

采用SPSS 17.0统计软件进行数据分析。CD82/KAI1、HIF-1α和VM表达阳性组与阴性组生存分析用*Kaplan-Meier*法, 组间比较用*Log-rank*检验, 多因素分析采用*Cox*回归多因素模型, 在NSCLC组织中, CD82/KAI1、HIF-1α的表达和VM与正常肺组织、各临床及病理因素的相关性采用*χ*^2^和*Spearman*等级相关检验, *P* < 0.05为差异有统计学意义。

## 2 结果

### 2.1 NSCLC中存在VM

HE染色光镜下可见由肿瘤细胞围成的管道样结构, 无内皮细胞衬覆, 管腔内不见坏死的肿瘤细胞及炎症细胞。经CD34与PAS双重染色, 管腔呈现CD34阴性但PAS阳性的结构, 即VM(图 1A, 图 1B); 此外, 还能见到网络状的VM。160例NSCLC标本中, 36.9%(59/160)可见由CD34阴性的肿瘤细胞围成的管道样或网络状的结构, 而PAS阳性, 一层PAS阳性物质将肿瘤细胞和管腔分开。正常肺组织中未见有VM现象。

**1:**
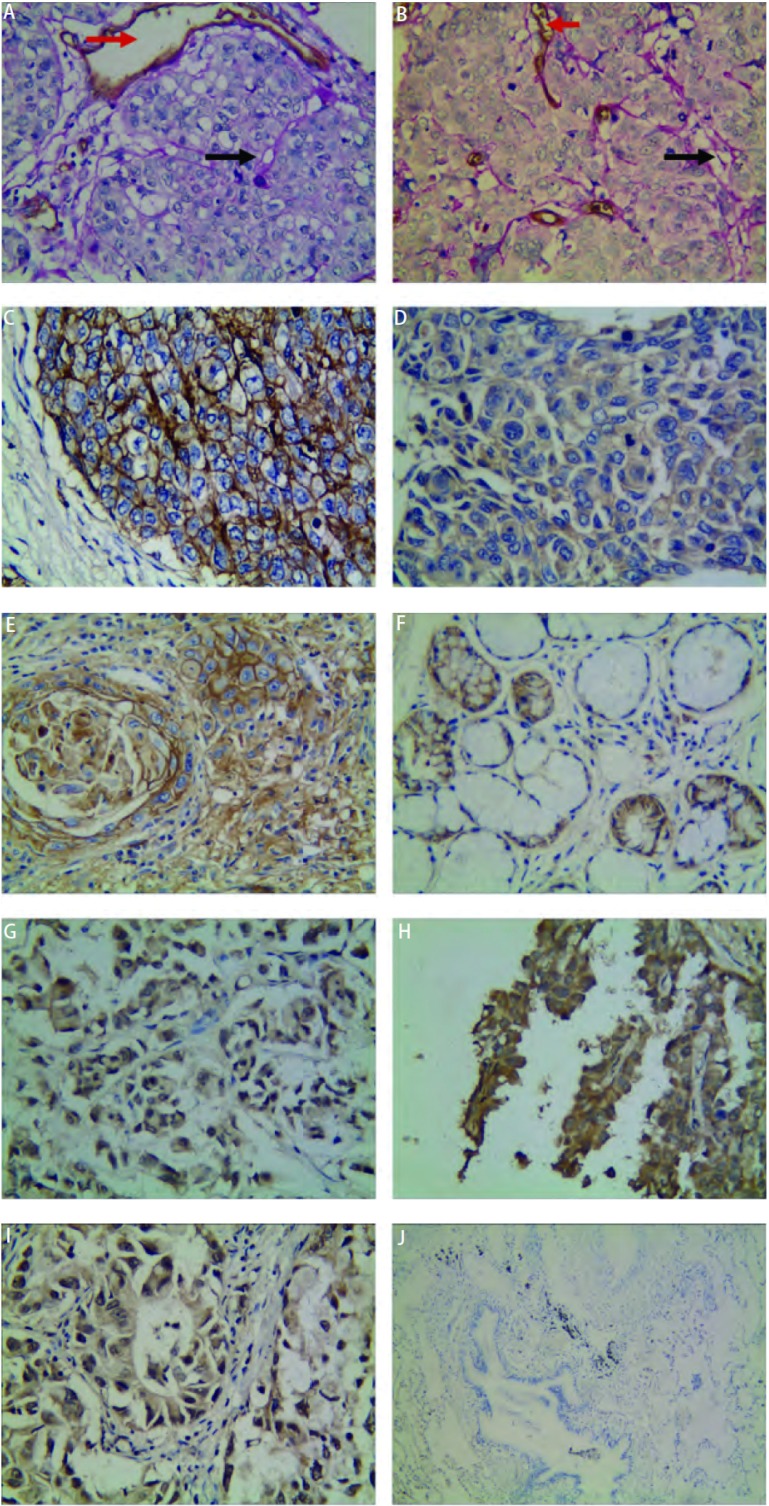
VM、CD82/KAI1和HIF-1*α*在NSCLC中的阳性染色以及对照组中的染色。A、B:NSCLC中VM阳性染色(黑箭头所示为VM结构, 红箭头所示为血管, A和B为低分化鳞癌, Elivision^TM^×400);C、D、E:NSCLC中CD82/KAI1表达阳性, 主要表达于细胞膜和细胞浆(C为中分化鳞癌, D为低分化鳞癌, E为高分化鳞癌, Elivision^TM^×400);F:对照组中CD82/KAI1表达阳性, 主要表达于细胞浆和细胞膜(为正常支气管腺, Elivision^TM^×400);G、H、I:NSCLC中HIF-1*α*表达阳性, 主要表达于细胞核和细胞浆(G为低分化腺癌, H为中分化鳞癌, I为中分化腺癌, Elivision^TM^×400);J:对照组中HIF-1*α*表达阴性(正常肺组织, Elivision^TM^×100)。 Positive staining of VM, CD82/KAI1 and HIF-1*α* in NSCLC and staining in the control group.A, B:positive staining of VM in NSCLC (black arrow is VM, red arrow is vessel, A and B are poor differentiation squamous carcinoma, Elivision^TM^×400);C, D, E:positive staining of CD82/KAI1 in membrane and plasma of NSCLC (C is mediate differentiation squamous carcinoma, D is poor differentiation squamous carcinoma, E is well differentiation squamous carcinoma, Elivision^TM^×400);F:positive staining of CD82/KAI1 in plasma and membrane of the control group (normal bronchial glands, Elivision^TM^×400);G, H, I:positive staining of HIF-1*α* in nuclei and plasma of NSCLC (G is poor differentiation adenocarcinoma, H is mediate differentiation squamous carcinoma, I is mediate differentiation adenocarcinoma, Elivision^TM^×400);J:negative staining of HIF-1*α* in the control group (J is normal lung tissue, Elivision^TM^×100).VM:vasculogenic mimicry; HIF-1*α*:hypoxia inducible factor-1*α*; NSCLC:non-small cell lung cancer.

### 2.2 NSCLC中的VM与临床病理的关系

VM阳性在患者的性别、年龄、组织学类型等之间的差异无统计学意义(*P* > 0.05)。随着NSCLC的分化越差, VM的阳性越强, 差异有统计学意义(*P* < 0.01), pTNM分期Ⅰ期-Ⅱ期VM阳性率为4.1%(3/74), Ⅲ期-Ⅳ期VM阳性率为65.1%(56/86), VM组的pTNM分期与无VM组的pTNM分期相比差异有统计学意义(*P* < 0.01), 且VM组的患者更易发生淋巴结转移(*P* < 0.01)(表 1)。

**1:** NSCLC中VM、CD82/KAI1、HIF-1*α*和MVD的表达与临床病理因素的关系 Correlation of VM and CD82/KAI1 and HIF-1*α* and MVD expression to clinicopathologic characteristics in NSCLC

Variable	VM		*χ*^2^	CD82/KAI1		*χ*^2^	HIF-1*α*		*χ*^2^	MVD		*χ*^2^
	-	+		-	+		-	+		< 22	≥22	
Gender			1.521			1.849			3.143			4.287^*^
Male	85	45		78	52		71	59		79	51	
Female	16	14		22	8		11	19		12	18	
Age (yr)			0.317			0.168			0.433			0.870
< 60	45	29		45	29		40	34		45	29	
≥60	56	30		55	31		42	44		46	40	
Gross type			2.014			0.569			0.834			0.008
Central	72	48		73	47		59	61		68	52	
Peripheral	29	11		27	13		23	17		23	17	
Histological type			0.424			0.301			0.038			1.170
Squamous cell carcinoma	75	41		71	45		60	56		69	47	
Adenocarcinoma	26	18		29	15		22	22		22	22	
Diameter (cm)			0.069			0.044			0.139			0.066
< 3.0	9	6		9	6		7	8		9	6	
≥3.0	92	53		91	54		75	70		82	63	
Differentiation			33.816^#^			33.944^#^			19.128^#^			20.066^#^
Well	20	1		2	19		19	2		19	2	
Mediate	72	31		67	36		52	51		61	42	
Poor	9	27		31	5		11	25		11	25	
Lymph node metastasis			31.025^#^			14.142^#^			36.023^#^			30.318^#^
Yes	31	45		41	43		20	56		26	50	
No	70	14		59	17		62	22		65	19	
PTNM stage			63.712^#^			48.439^#^			97.173^#^			85.686^#^
Ⅰ-Ⅱ	71	3		25	49		69	5		71	3	
Ⅲ-Ⅳ	30	56		75	11		13	73		20	66	
^*^P < 0.05, ^#^*P* < 0.01.												

### 2.3 NSCLC中CD82/KAI1的表达及其与临床病理的关系

CD82/KAI1主要定位于NSCLC癌细胞膜, 胞浆也有一定表达, 呈棕黄色。正常肺组织中CD82/KAI1阳性颗粒主要位于细胞浆和膜(图 1F)。在NSCLC中, CD82/KAI1的阳性率为37.5%(图 1C, 图 1D, 图 1E), 较正常肺组织的表达率(19/20)明显减少(*P* < 0.01)。CD82/KAI1的表达与患者性别、年龄及组织学类型无相关性(*P* > 0.05)。随着NSCLC的分化越差, CD82/KAI1的表达越低, 差异有统计学意义(*P* < 0.01);CD82/KAI1的阳性表达与淋巴结转移有关(*P* < 0.01);Ⅰ期-Ⅱ期肿瘤中CD82/KAI1的阳性率为66.2%(49/74), Ⅲ期-Ⅳ期肿瘤中CD82/KAI1的阳性率为12.8%(11/86), 两者之间差异具有统计学意义(*P* < 0.01)(表 1)。

### 2.4 NSCLC中HIF-1α的表达及其与临床病理的关系

HIF-1α主要表达于NSCLC癌细胞核, 棕黄色, 在胞浆中也有少量表达。对照组未见有HIF-1α表达(图 1J), 在NSCLC中HIF-1α蛋白阳性表达率为48.8%(图 1G, 图 1H, 图 1I), 其表达与NSCLC的分化程度、淋巴结转移及临床分期有关, 即随着HIF-1α表达越高, 肿瘤的分化越差、临床分期越晚、更易发生淋巴结转移(表 1)。

### 2.5 NSCLC中CD82/KAI1、HIF-1α的表达和VM情况以及它们的关系

在VM阳性组的病例中, CD82/KAI1阳性率为3.4%(2/59);在CD82/KAI1阳性表达的病例中, VM的阴性率为96.7%(58/60), 差异有统计学意义。Spearman相关分析显示, CD82/KAI1在NSCLC中的表达与VM呈负相关(*r*=-0.539, *P* < 0.01)。从表 2可以看出, CD82/KAI1与HIF-1α的表达呈负相关(*r*=-0.704, *P* < 0.01), HIF-1α的表达与VM呈正相关(*r*=0.654, *P* < 0.01)。

**2:** NSCLC中VM、CD82/KAI1、HIF-1α和MVD各因素之间的相互关系 The expression of VM and CD82/KAI1 and HIF-1α and MVD, and their relationship in NSCLC

Variable	CD82/KAI1		*r*	HIF-1*α*		*r*	MVD		*r*
	N	P		N	P		< 22	≥22	
VM			-0.539^*^			0.654^#^			0.747^#^
Negative	43	58		77	24		86	15	
Positive	57	2		5	54		5	54	
CD82/KAI1						-0.704^*^			-0.596^*^
Negative	-	-		24	76		34	66	
Positive	-	-		58	2		57	3	
HIF-1*α*			-0.704^*^						0.716^#^
Negative	24	58		-	-		75	7	
Positive	76	2		-	-		16	62	
N:negative; P:positive; ^*^is negative corelation; ^#^is positive correlation, *P* < 0.01.									

### 2.6 MVD与CD82/KAI1、HIF-1α、VM的关系

*Spearman*相关分析显示, CD82/KAI1的表达与MVD呈负相关(*r*=-0.596, *P* < 0.01);VM的阳性与MVD呈正相关(*r*=0.747, *P* < 0.01);HIF-1α的表达与MVD呈正相关(*r*=0.716, *P* < 0.01)(表 2)。

### 2.7 多因素分析

将病理组织分化(分为高分化组、中分化组与低分化组), 年龄(分为≥60岁组与 < 60岁组)、pTNM分期(分为Ⅰ期+Ⅱ期组与Ⅲ期+Ⅳ期组)、肿瘤直径(分为≥3.0 cm组与 < 3.0 cm组)、肿瘤位置(分为中央型组与周围型组)、淋巴结转移(分为有转移组与无转移组)、性别(男性组与女性组)、VM(分为阳性组与阴性组)、MVD(因为MVD的均值为22.42±12.8, 故以MVD < 22为阴性, MVD≥22为阳性); CD82/KAI1(分为表达阳性组与阴性组)和HIF-1α(分为表达阳性组与阴性组)等因素引入*Cox*模型进行多因素分析, 结果显示:CD82/KAI1与HIF-1α的表达和VM及pTNM分期是影响NSCLC患者预后的独立因素(表 3)。

**3:** 160例NSCLC患者多因素分析 Multivariate survival analysis of 160 patients with NSCLC

Covariate	*B*	*SE*	*Wald*	*df*	*Sig*	*Exp (B)*	95%CI
pTNM	1.095	0.400	7.494	1	0.006	2.990	1.365-6.549
VM	0.933	0.296	9.907	1	0.002	2.542	1.422-4.543
CD82/KAI1	-1.489	0.292	26.036	1	0.000	0.226	0.127-0.400
HIF-1*α*	0.842	0.323	6.815	1	0.009	2.322	1.234-4.371
MVD	-0.263	0.312	0.709	1	0.395	0.400	0.417-1.418
^*^MVD≥22 is positive, MVD < 22 is negative.							

### 2.8 生存分析

本组病例总的5年生存率为26.9%。*Kaplan-Meier*生存分析显示VM阳性组与阴性组5年生存率分别为1.7%(1/59)和41.6%(42/101), 差异有统计学意义(*P* < 0.01, 图 2A); CD82/KAI1阳性组与阴性组5年生存率分别为68.3%(41/60)和2.0%(2/100), 差异有统计学意义(*P* < 0.01, 图 2B); HIF-1α阳性组与阴性组5年生存率分别为3.8%(3/78)和48.8%(40/82), 差异有统计学意义(*P* < 0.01, 图 2C); MVD≥22和MVD < 22的5年生存率分别为2.9%(2/69)和45.1%(41/91), 差异有统计学意义(*P* < 0.01, 图 2D)。

**2:**
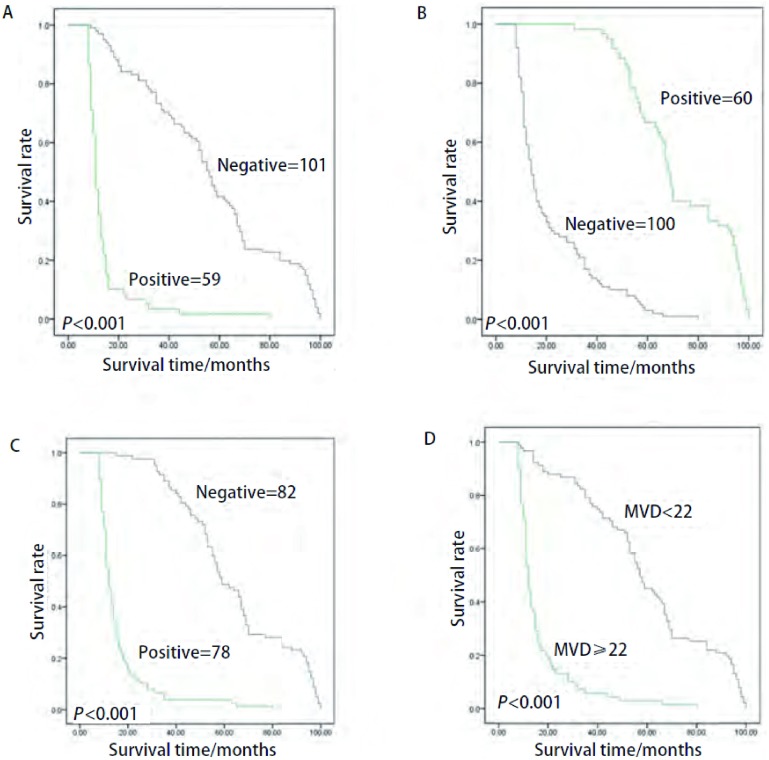
VM、CD82/KAI1、HIF-1*α*和MVD阳性组与阴性组NSCLC患者生存曲线。A:VM阳性组与阴性组NSCLC患者生存曲线; B:CD82/KAI1表达阳性组与阴性组NSCLC患者生存曲线; C:HIF-1*α*表达阳性组与阴性组NSCLC患者生存曲线; D:MVD≥22组与 < 22组NSCLC患者生存曲线。MVD:microvessel density。 Survival curves of NSCLC patients with positive or negative group VM, CD82/KAI1, HIF-1*α* and MVD.A:survival curves of NSCLC patients with positive or negative VM group; B:survival curves of NSCLC patients with positive or negative CD82/KAI1 expression; C:survival curves of NSCLC patients with positive or negative HIF-1αexpression; D:survival curves of NSCLC patients with MVD≥22 group or < 22 group.

## 3 讨论

部分高侵袭性肿瘤存在一种不依赖血管内皮细胞, 而是由肿瘤细胞通过自身变形和细胞外基质重塑直接围成的管道样结构, 并可与宿主静脉相通, 以获取血供, 即血管生成拟态-VM^[5]^。VM的提出不仅对传统的血管生成理论提出了挑战, 同时也是对肿瘤血管形成理论的重要补充。Folberg等^[6]^在葡萄膜黑色素瘤中定义了几种形态的VM, 即有的呈直的或平行排列的或十字交叉的直线型、有或无分支的弧形、封闭的环形、网络状等结构, 这些结构都呈现PAS阳性而CD34阴性。后来在肝癌^[7]^、卵巢癌^[8]^、前列腺癌^[9]^及双分化肿瘤^[10]^等高度恶性肿瘤中都报道有VM的存在。

本研究在59例NSCLC中发现了符合具有VM标准的结构, 证实NSCLC中有VM的存在。VM结构由肿瘤细胞围成, 肿瘤细胞与血流之间仅有一层PAS阳性物质相隔, 无血管内皮细胞屏障, 导致了肿瘤患者具有转移早、转移率高、患者临床预后差, 死亡率高等特点。本研究也显示VM与肿瘤分化程度、淋巴结转移与否及临床分期等密切相关。即有VM的肿瘤具有更差的分化、更低的临床分期以及更易发生淋巴结转移。

*HIF-1α*基因位于14号染色体, 编码826个氨基酸。当细胞缺氧时, HIF-1α在细胞核内高表达。HIF-1α可通过调控多种靶基因表达以逃避或适应相对缺氧环境, 参与肿瘤生长、浸润和转移^[11]^。本研究结果显示, HIF-1α表达高的肿瘤, 具有分化差、临床分期晚和易发生淋巴结转移等特点。且HIF-1α和MVD的表达及VM相互之间具有正相关性, 这说明缺氧可能会参与肿瘤的血管与VM的形成。缺血和缺氧是恶性肿瘤发展过程中的普遍现象, 当肿瘤细胞缺氧时, HIF-1α会上调VEGF表达^[12]^, 诱导新生血管形成, 可以暂时缓解肿瘤细胞的缺血、缺氧状态。当新生血管的血供还不能够满足肿瘤细胞营养需求时, 这时某些具有可塑性的肿瘤细胞就会变形, 形成具有VM形态的结构以获得足够供氧, 所以具有VM结构的肿瘤细胞周围没有坏死。本研究结果与国内外文献^[13-16]^报道一致。同时文献^[15]^还报道, 抑制HIF-1α的表达可以阻断VM的形成, 这说明缺氧很可能是诱导肿瘤细胞形成VM的一个重要始动因素。

*CD82*/*KAI1*基因首先发现于前列腺癌细胞, 曾被认为是前列腺癌特异性的转移抑制基因, 命名为*KAI1*基因^[17]^, 后来对该基因的定位及DNA全长序列测序证明, 其与*CD82*基因完全相同, 故又将其命名为*CD82*/*KAI1*基因, 属于TM4SF家族。在肿瘤中*CD82*/*KAI1*基因的异常形式表现为基因突变、等位基因缺失和表达水平的改变等, 其中表达水平与肿瘤侵袭、转移关系密切。本研究发现CD82/KAI1蛋白在肿瘤组织中的表达水平比在正常组织中明显降低, 差异具有统计学意义; 并且随着肿瘤分化越低、临床分期越晚, 其表达水平也越低, 差异有统计学意义; 在伴有淋巴结转移的肿瘤中其表达水平也较没有淋巴结转移的肿瘤明显降低, 与文献^[18, 19]^报道一致。提示*CD82*/*KAI1*基因表达异常与NSCLC的发生、发展及侵袭、转移密切相关。CD82/KAI1表达异常使CD82/KAI1正常功能减弱或丧失, 从而失去抑制肿瘤转移的功能。

本研究多因素分析显示HIF-1α和CD82/KAI1的表达情况、VM和pTNM分期是影响NSCLC的独立预后因素, 而MVD并非NSCLC的独立预后因素。pTNM分期作为术后治疗的标准已为人们所重视, 因此寻找新的可以反映肿瘤细胞生物学行为并对pTNM分期起到一定补充作用的分子标记物显得更为重要。进一步的生存分析显示VM、HIF-1α和MVD阳性组的5年生存率明显低于阴性组, 提示VM、HIF-1α和MVD阳性组的NSCLC患者的生存时间比较短, 比其阴性组预后差, 这与文献^[21, 22]^报道一致。CD82/KAI1阳性组与阴性组的5年生存率分别为66.7%和3.0%, 差异有统计学意义, 即CD82/KAI1阳性表达组的生存时间高于阴性表达组, 这与其他学者研究结果相同^[19-20]^。

本研究中, VM和HIF-1α在对照组20例正常肺组织中均不存在, 而在NSCLC中表达于部分肺癌细胞, 差异明显; 并且随着肿瘤的进展, VM、HIF-1α和MVD的阳性率增多。对CD82/KAI1蛋白与VM、HIF-1α、MVD的表达进行相关性分析, 发现癌组织中随着CD82/KAI1表达率的降低, VM、HIF-1α和MVD的阳性率明显增多, 呈负相关。提示CD82/KAI1蛋白的表达与VM、HIF-1α、MVD的阳性率可能存在一定联系。随着肿瘤的快速进展, 肿瘤组织会发生缺血缺氧, 缺氧会诱导新生血管及VM形成; 而此时CD82/KAI1表达降低导致癌细胞间的粘附力减弱, 移动性增强、细胞分化低^[20]^; 而具有VM结构的肿瘤细胞与管腔仅间隔一层PAS阳性物质, 此时粘附力降低的肿瘤细胞在血流的冲击下, 很容易脱离原发灶而发生淋巴结转移甚至远处转移^[20]^。

综上所述, *CD82*/*KAI1*基因表达降低可能是NSCLC发生侵袭、转移的分子基础, 血管生成和血管生成拟态可能是NSCLC发生侵袭、转移的关键事件。因此, CD82/KAI1、VM、HIF-1α和MVD可作为评估NSCLC转移和预后的指标。

## References

[1] Muneyuki T, Watanabe M, Yamanaka M (2001). KAI1/CD82 expression as a prognostic factor in sporadic colorectal cancer. Anticancer Res.

[2] Gellersen B, Briese J, Oberndörfer M (2007). Expression of the metastasis suppressor KAI1 in decidual cells at the human maternal-fetal interface:Regulation and functional implications. Am J Pathol.

[3] Yue WY, Chen ZP (2005). Does vasculogenic mimicry exist in astrocytoma?. J Histochem Cytochem.

[4] Kumada T, Tsuneyama K, Hatta H (2004). Improved 1-h rapid immunostaining method using intermittent microwave irradiation:practicability based on 5 years application in Toyama Medical and Pharmaceutical University Hospital. Mod Pathol.

[5] Vartanian A, Baryshnikov AY (2007). Crosstalk between apoptosis and antioxidants in melanoma vasculogenic mimicry. Adv Exp Med Biol.

[6] Folberg R, Maniotis AJ (2004). Vasculogenic mimicry. APMIS.

[7] Zhao XL, Du J, Zhang SW (2006). A study on vasculogenic mimicry in hepatocellular carcinoma. Chin J Hepatol.

[8] Sood AK, Seftor EA, Fletcher MS (2001). Molecular determinants of ovarian cancer plasticity. Am J Pathol.

[9] Danny R, Gray I, Wendy J (2004). Short-term human prostate primary xenografts:an *in vivo* model of human prostate cancer vasculature and angiogenesis. Cancer Res.

[10] Sun BC, Zhang SW, Ni CS (2005). The clinical significance study of vasculogenetic mimicry in 337 cases of Bi-directional differential malignant tumors. Chin J Clin Oncol.

[11] Swinson DE, Jones JL, Cox G (2004). Hypoxia-inducible factor-1 alpha in non small cell lung cancer:elation to growth factor, protease and apoptosis pathways. Int J Cancer.

[12] Liu LX, Lu H, Luo Y (2002). Stabilization of vascular endothelial growth factor mRNA by hypoxia-inducible factor 1. Biochem Biophys Res Commun.

[13] Sun B, Zhang D, Zhang S (2007). Hypoxia influences vasculogenic mimicry channel formation and tumor invasion-related protein expression in melanoma. Cancer Lett.

[14] Li M, Gu Y, Zhang Z (2010). Vasculogenic mimicry:a new prognostic sign of gastric adenocarcinoma. Pathol Oncol Res.

[15] Yao LQ, Feng YJ, Ding JX (2005). Primary study of vasculogenic mimicry induced by hypoxia in epithelial ovarian carcinoma. Zhonghua Fuchanke Zazhi.

[16] Hendrix MJ, Seftor RE, Seftor EA (2002). Transendothelial function of human metastatic melanoma cells:role of the microenvironment in cell-fate determination. Cancer Res.

[17] Dong JT, Lamb PW, Rinker Schaeffer CW (1995). KAI1, a metastasis suppressor gene for prostate cancer on human chromosome 11P11.2. Science.

[18] Takeda TY, Hattori N, Tokuhara T (2007). Adenoviral transduction of MRP-1/CD9 and KAI1/CD82 inhibits lymph node metastasis in orthotopic lung cancer model. Cancer Res.

[19] Zheng HC, Tsuneyama K, Cheng C (2007). Expression of KAI1 and tenascin, and microvessel density are closely correlated with liver metastasis of gastrointestinal adenocarcinoma. J Clin Pathol.

[20] Xu XJ, Mei TH (2010). Expression of KAI1 in non-small cell lung cancer and its relationship between to metastasis and prognosis. Mod Pract Med.

[21] Shirakawa K, Wakasugi H, Heike Y (2002). Vasculogenic mimicry and pseudocomedo formation in breast cancer. Int J Cancer.

[22] Bos R, Vander Groep P, Greijer AE (2003). Levels of hypoxia-inducible factor-1alpha independently predict prognosis in patients with lymph node negative breast carcinoma. Cancer.

